# Safety of penicillamine and trientine in the treatment of Wilson’s disease: An analysis of the FDA Adverse Event Reporting System (FAERS) database

**DOI:** 10.1371/journal.pone.0336721

**Published:** 2025-11-12

**Authors:** Wenlong Qian, Kou Xu, Shuo Li, Zhuo Zhang, Xiaoxiao Hou, Bingjie Min, Jia Ling, Xinyu Zhu, Hui Zhou, Wenjuan Xu, Wenming Yang, Shijian Cao, Yonghua Chen

**Affiliations:** 1 Department of Neurology, The First Affiliated Hospital of Anhui University of Chinese Medicine, Hefei, Anhui, China; 2 Center for Xin’an Medicine and Modernization of Traditional Chinese Medicine, Institute of Health and Medicine Hefei Comprehensive National Science Center, Hefei, Anhui, China; 3 Key Laboratory of Xin’An Medicine, Ministry of Education, Anhui, China; Peking University First Hospital, CHINA

## Abstract

**Background:**

Penicillamine(D-Penicillamine) and trientine are first-line therapies for Wilson’s Disease (WD), yet real-world data on their adverse events (AEs) remain scarce. We analyzed the FDA Adverse Event Reporting System (FAERS) to comprehensively assess the safety of penicillamine and trientine in WD treatment.

**Methods:**

AEs for penicillamine and trientine (2004Q1–2024Q4) were analyzed using Proportional Reporting Ratio (PRR), Reporting Odds Ratio (ROR), and Bayesian Confidence Propagation Neural Network (BCPNN).

**Results:**

We found 1,452 and 760 AEs related to penicillamine and trientine, respectively. In all adverse event (AE) reports, the ratio of females to males was approximately 1.3, with the highest proportion of AE reports in the 21–30 age group, and the largest number of AE reports coming from the United States. Signal detection showed that the most commonly reported AEs for penicillamine and trientine were drug hypersensitivity and tremor, respectively, with the highest proportions in the SOC categories of immune system disorders and gastrointestinal disorders. The main AEs for both drugs involved condition aggravated, and identified potential safety signals requiring further validation for the two drugs, such as decreased bone density and brain atrophy for penicillamine, and memory impairment, oesophageal ulcer and starvation for trientine. In addition, we found that women were more likely to experience drug hypersensitivity in penicillamine adverse event reports, while men were more likely to experience cutis laxa.

**Conclusion:**

This study reveals the characteristics of AEs and potential associated risks in the clinical application of penicillamine and trientine, emphasizing individualized medication and vigilant monitoring strategies to provide guidance for safe medication use.

## Introduction

Wilson Disease (WD) is an autosomal recessive disorder caused by mutations in the ATP7B gene on chromosome 13, resulting in defective copper transport [1]. The ATP7B protein, predominantly expressed in hepatocytes, facilitates copper incorporation into ceruloplasmin and biliary copper excretion [2]. Mutations in ATP7B disrupt these processes, leading to pathological copper accumulation in the liver, basal ganglia, and cornea. This accumulation induces hepatocellular necrosis, astrocyte dysfunction, and oxidative stress-mediated damage, ultimately progressing to cirrhosis, neuropsychiatric manifestations (e.g., tremor, dysarthria), and Kayser-Fleischer rings [3]. Globally, WD affects approximately 1 in 30,000 individuals, with heterozygous carrier rates reaching 1 in 90 in East Asia [2]. It is also noteworthy that the direct sequencing of the ATP7B gene has transitioned from being a diagnostic aid to a core test for WD. Along with biochemical assays and population genetics, this shift has led to the realization that the disease’s prevalence could have been underestimated [4].

Penicillamine (clinically known as D-Penicillamine) was first proposed for WD treatment by Walshe in 1956 and has since become a mainstay intervention for the disease [5]. Both the 2025 European Association for the Study of the Liver (EASL) [6] and American Association for the Study of Liver Disease (AASLD) [3] guidelines recommend penicillamine as the drug of choice for WD treatment. Mechanistically, penicillamine binds to free copper through its sulfhydryl group, forming a soluble complex, which in turn, promotes urinary copper excretion. In other words, penicillamine could effectively reduce copper accumulation in the liver, lowering the hepatic copper load in patients [5]. Nonetheless, its clinical utility is limited by a high incidence of adverse events (AEs; 30–50%), including myelosuppression, nephrotic syndrome, and immune-mediated complications, often necessitating discontinuation [7]. To overcome penicillamine’s prominent AEs, trientine was approved as an alternative second-line intervention in 1969. Besides being more selective in copper chelation by binding it and promoting its excretion through the kidneys, trientine could also inhibit the intestinal absorption of copper, thus decreasing its transport in the portal vein and hepatic uptake. Trientine has also been linked with fewer immune reactions [8,9]. Despite improved tolerability, trientine carries risks of hepatic enzyme abnormalities, neurological deterioration, and gastrointestinal (GI) AEs [10–12]. The current understanding of the safety of penicillamine and trientine is primarily informed by small-sample cohort studies or single-center retrospective analyses. A retrospective study encompassing 380 WD patients reported a significantly higher rate of Adverse events (AEs) leading to discontinuation in the penicillamine group than in the trientine group (P = 0.039), highlighting tolerability differences [7].

The U.S. FDA Adverse Event Reporting System (FAERS) database, a cornerstone of post-marketing pharmacovigilance, aggregates spontaneous AE reports from healthcare professionals, patients, and manufacturers worldwide [13]. Through data mining techniques [e.g., Proportional Reporting Ratios (PPRs) and Bayesian Confidence Propagation Neural Networks (BCPNN), among others], researchers could identify potential AE signals and quantify the risk profiles of different drugs. While Kumar et al. analyzed penicillamine AEs in FAERS (1970–2020) [14], no study has systematically compared AE profiles of penicillamine and trientine using this resource. Additionally, Schilsky et al. [15] conducted a randomized, open-label, non-inferiority, phase 3 trial to compare the safety and efficacy of penicillamine to that of tetracycline hydrochloride for maintenance therapy in WD patients. According to the results, penicillamine correlated with three post-randomization Severe Adverse Events [SAEs; leukopenia, cholangiocarcinoma, and hepatocellular carcinoma], whereas no such events were reported for TETA4. Given the lifelong therapy requirement for WD and the critical need to balance efficacy with AE management, we analyzed AE reports from 2004Q1-2024Q4 in the FAERS database to comprehensively compare the AE profiles of penicillamine and trientine.Unlike previous studies that focused solely on penicillamine or small cohort comparisons, our analysis is the first to systematically compare the two drugs over a 20-year period and uses three signal detection methods to enhance reliability, filling a gap in the WD literature that lacks comparative pharmacovigilance data.This study aims to analyze data from FAERS from 2004 to 2024 to compare the AE characteristics of penicillamine and trientine, identify potential safety signals associated with each drug by applying multiple algorithms, and explore gender differences in the risk of AEs. We hypothesized that these two drugs would exhibit distinct AE spectra, with penicillamine being more likely to cause immune-related AEs and trientine being more prone to inducing gastrointestinal events.This study provides a basis for clinical decision-making in WD treatment and a reference for the research and development of safer and more selective copper chelating agents,such as tetrasulfomolybdate [16].

## Methods

### Study design and data sources

This retrospective study explored real-time data on AEs associated with penicillamine and trientine. The primary data source was the U.S. FAERS, the platform’s openness and real-time nature offer a unique advantage for drug safety research [17,18]. It collects AE reports submitted by healthcare professionals, consumers, and manufacturers worldwide, enabling the detection of safety signals for approved drugs. The database contains seven standardized datasets, including demographic information, drug details, adverse events, patient outcomes, report sources, treatment dates, and usage indications [19]. As a voluntary reporting system, FAERS has limitations such as underreporting and reporting bias; however, it remains a valuable resource for identifying potential drug-related adverse events in real-world settings. To better represent the current status of the drugs’ AEs in the real world, our data extraction was limited to 20 years of publicly available reports from the first quarter of 2004 through to the fourth quarter of 2024. The resulting dataset comprised seven tables: Demographics (DEMO), Drug (DRUG), Reporting Source (RPSR), Treatment (THER), Indication (INDI), Response (REAC), and Outcome (OUTC). The target drugs were penicillamine and trientine. To ensure search completeness, their generic names, trade names, and common misspellings—such as “Penicilamine” and D-penicillamine for Penicillamine and “Trientine HCl” for Trientine—were combined and coded uniformly. Only reports in which the target drug was the “Primary Suspect” were included and those in which the target drugs were “Concomitant” or “Interacting” compounds were excluded. The PRIMARYID, CASEID, and FDA_DT fields in the DEMO table were sorted in ascending order by CASEID, FDA_DT, and PRIMARYID, respectively. Due to the nature of data updates, duplicate reports are inevitable in FAERS. To ensure data integrity, we deduplicated adverse event reports following FDA-recommended procedures [20]. Specifically, for reports sharing the same CASEID, we retained the record with the latest FDA_DT. When multiple reports shared identical CASEID and FDA_DT, we selected the report with the highest PRIMARYID. Subsequently, we categorized and described AEs based on Preferred Terminologies (PTs) and System Organ Classes (SOCs) as outlined in the International Medical Dictionary of Regulatory Activities (MedDRA version 27.0). Entire case reports were used for analysis, contribution to the results of the analysis was considered if at least one of the case reports contained a suspect drug-induced AE. Requirements for ethical approval were waived due to the public availability of the data sources.

### Statistical analysis

Disproportional Analysis (DPA), a commonly used data mining method, encompasses Reporting Odds Ratio (ROR), PRR, BCPNN, and Multi-Item Gamma Poisson Shrinker (MGPS) algorithms. Although PRR [21] is commonly used to estimate relative risk, it is sensitive and prone to false positive signals. On the other hand, ROR [22] provided consistent estimates of ratios and risk ratios, with less bias relative to other indices. Furthermore, BCPNN [23] employed a supervised neural network approach, using known AEs as a Machine Learning (ML) training set, resulting in a higher specificity and a stronger correlation in the detection of Adverse Reactions (ARs). Finally, MGPS [24] effectively balanced data noise and signal via Bayesian contraction, offering a more suitable approach for analyzing high-dimensional sparse data.Empirical Bayes Geometric Mean (EBGM), a measure derived from MGPS, was not employed because ROR, PRR, and BCPNN collectively provide robust sensitivity-specificity balance, and EBGM may over-shrink signals in sparse data contexts.Here, we combined the ROR, PRR, and BCPNN methods to mine the target drugs’ AE signals.The signal was suspected of an AE when the results of all three methods were positive. [25] We only reported signals detected by all three methods to balance specificity (reducing false positives) and sensitivity, acknowledging this may miss weak signals detected by single methods. Table 1 details the measurements and criteria for signal detection.

**Table 1:** Formula and signal detection criteria for reporting odds ratio (ROR), proportional reporting ratio (PRR) and Bayesian confidence propagation neural network (BCPNN).

Algorithms	Equation	Criteria	
ROR	ROR = ad/bc 95%CI = eIn(ROR)±1.96(1/a + 1/b + 1/c + 1/d)^0.5	Lower limit of 95%CI > 1,a ≥ 3	
PRR	PRR = a(c + d)/c/(a + b) χ²=[(ad-bc)^2](a + b + c + d)/[(a + b)(c + d)(a + c)(b + d)]	PRR > 2,χ² > 4,a ≥ 3	
BCPNN	IC = log2a(a + b + c + d)(a + c)(a + b) 95%CI = E(IC)±2V(IC)^0.5	IC025 ＞ 0	
Fourfold table of disproportionality measures.	Target AE	OtherAE	Total
Target drugs	a	b	a + b
Other drugs	c	d	c + d
Total	a + c	b + d	a + b + c + d

ROR reporting odds ratio; CI, confidence interval; PRR, proportional reporting ratio; χ2, chi-squared; BCPNN, bayesian confidence propagation neural network; IC, information component; IC025, the lower limit of 95%CI, of the IC.

Data analyses and plotting were performed using Microsoft Office Excel 2021, R 4.4.3 software, GraphPad Prism 9.5 software, and online plotting platforms (https://www.bioincloud.tech/task-meta) (https://www.bioinformatics.com.cn).

## Results

### Demographic characteristics

**Fig 1:**
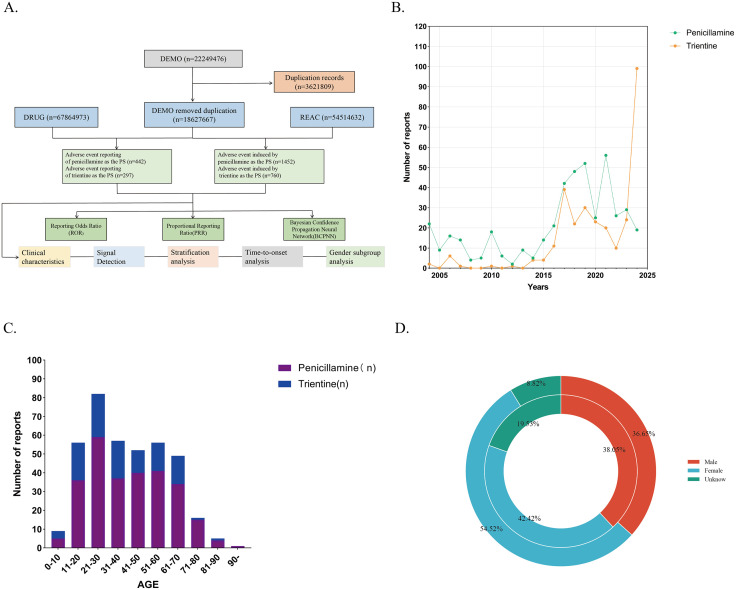
Demographic information reported by AEs in FAERS. **A** A flow chart of this study; **B** Number of two drugs reported per year; **C** Number of reports by age for the two drugs; **D** Rates were reported by sex for the two drugs;The outer circle represents penicillamine, and the inner circle represents trientine.

**Fig 2:**
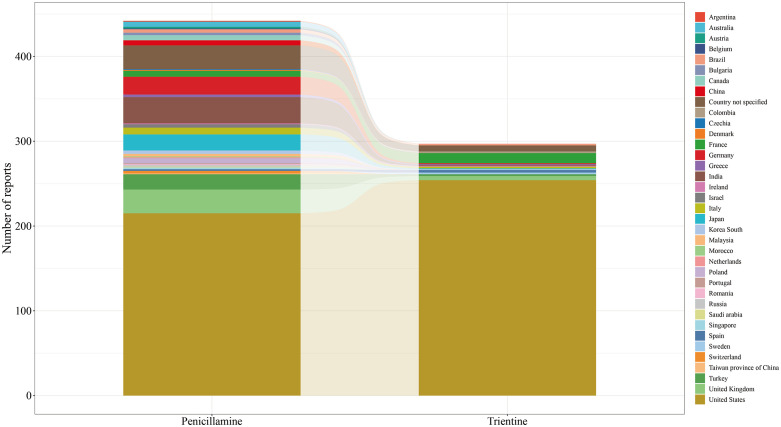
Number of reports by region for the two drugs.

After cleaning and de-duplication of the data acquired from 2004Q1 to 2024Q4, a total of 442 AE reports and 1,452 AEs were collected for “penicillamine” as the primary suspect (PS) drug, and 297 AE reports and 760 AEs were collected for trientine (Fig 1A). The overall number of AE reports for both drugs showed an increasing trend over the twenty-year period from 2004 to 2024, with the number of trientine AE reports peaking in 2024 (Fig 1B). Table 2 details the AE reporting characteristics. In terms of age, AE reports for penicillamine and trientine were concentrated in patients <65 years of age, with both drug-related AEs occurring more frequently in patients aged 21–30 years(Fig 1C).In terms of gender characteristics, the difference was more pronounced for penicillamine((Fig 1D), with a male-to-female ratio of 1:1.5.In terms of region of reporting, the majority of AEs for both penicillamine and trientine were reported in the United States (Fig 2), accounting for 48.6% and 85.5%, respectively. Notably, trientine was reported in Asia in only 2.0% of casesAmong the AE reports in the outcome data, serious outcomes (including hospitalization, disability, life-threatening, and death) associated with penicillamine and trientine, in addition to unknown and other outcomes, accounted for 33.7% and 26.6%, respectively. Of these, hospitalization was the most frequently reported serious outcome.

**Table 2:** AE-associated demographic information from FAERS.

Characteristics		Penicillamine(n = 442)	Trientine(n = 297)	
		n((%)	n(%)	
Sex	Female	241 (54.5%)	126 (42.4%)	
	Male	162 (36.7%)	113 (38.0%)	
	Unknown	39 (8.8%)	58 (19.5%)	
Weight(kg)	＜50	12 (2.7%)	7 (2.4%)	
	＞100	7 (1.6%)	5 (1.7%)	
	50 ～ 100	42 (9.5%)	36 (12.1%)	
	Unknown	381 (86.2%)	249 (83.8%)	
Age(yeas)	<20	41(9.3%)	26(8.8%)	
	>65	35(7.9%)	4(1.3%)	
	20 ～ 40	101(22.9%)	55(18.5%)	
	41 ～ 65	100(22.6%)	45(15.2%)	
	Unknown	165 (37.3%)	167(56.2%)	
Reporting year	2022-2024	74(16.7%)	133(44.8%)	
	2016-2021	244((55.2%)	145(48.8%)	
	2010-2015	54(12.3%)	10(3.4%)	
	2004-2009	70(15.8%)	9(3.0%)	
Outcome	Other	156(35.2%)	49(16.5%)	
	Hospitalization	83(18.8%)	44(14.8%)	
	Death	42(9.5%)	23 (7.7%)	
	Life- Threatening	14(3.2%)	2(0.7%)	
	Disability	10(2.3%)	10 (3.4%)	
	Unknown	137(31.0%)	169 (56.9%)	
Reported countries	Argentina	1(0.2%)	Brazil	2(0.7%)
	Australia	6(1.4%)	Colombia	2(0.7%)
	Austria	2(0.5%)	Country not specified	7(2.4%)
	Belgium	1(0.2%)	France	12(4.0%)
	Brazil	4(0.9%)	Germany	1(0.3%)
	Bulgaria	3(0.7%)	Greece	1(0.3%)
	Canada	6(1.4%)	India	1(0.3%)
	China	6(1.4%)	Ireland	1(0.3%)
	Country not specified	28(6.3%)	Italy	2(0.7%)
	Czechia	1(0.2%)	Japan	1(0.3%)
	Denmark	1(0.2%)	Korea, South	1(0.3%)
	France	7(1.6%)	Spain	3(1.0%)
	Germany	21(4.8%)	Sweden	2(0.7%)
	Greece	3(0.7%)	Turkey	2(0.7%)
	India	31(7.0%)	United Kingdom	5(1.7%)
	Ireland	1(0.2%)	United	254(85.5%)
	Israel	4(0.9%)	States	
	Italy	8(1.8%)		
	Japan	19(4.3%)		
	Korea, South	4(0.9%)		
	Malaysia	3(0.7%)		
	Morocco	1(0.2%)		
	Netherlands	1(0.2%)		
	Poland	6(1.4%)		
	Portugal	1(0.2%)		
	Romania	1(0.2%)		
	Russia	2(0.5%)		
	Saudi arabia	1(0.2%)		
	Singapore	2(0.5%)		
	Spain	2(0.5%)		
	Switzerland	3(0.7%)		
	Taiwan, province of China	1(0.2%)		
	Turkey	18(4.1%)		
	United Kingdom	28(6.3%)		
	United States	215(48.6%)		

### AE signal mining and analysis

**Fig 3:**
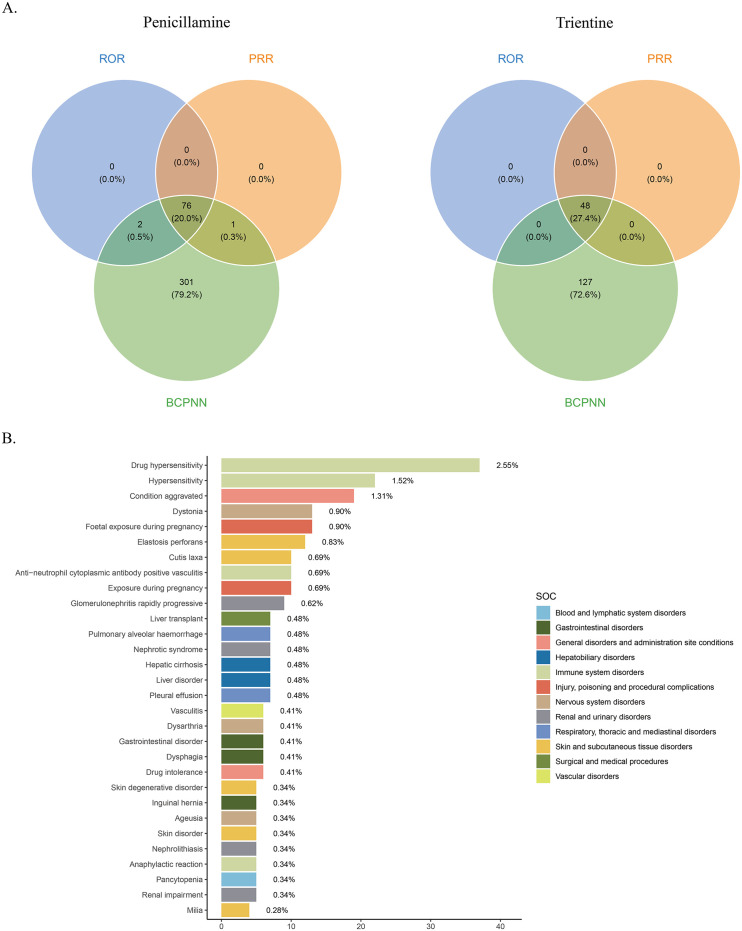
Signal intersection and signal distribution characteristics. A Positive signal intersection of three signal detection methods (ROR, PRR, BCPNN) for the two drugs. B Top 30 AE names and corresponding SOCs with the highest percentage of signals for penicillamine.See S1 Fig for Figure **C.**

Herein, AE signals were analyzed using ROR, PRR, and BCPNN at the Preferred Term (PT) level. The ROR method yielded 78 and 48 PT signals for penicillamine and trientine, respectively. Furthermore, the PRR method yielded 77 and 48 PT signals for penicillamine and trientine, respectively. On the other hand, the BCPNN method yielded 380 and 175 PT signals for penicillamine and trientine, respectively. Among all PT signals identified using the three algorithms, only 76 and 48 met the positive criteria for penicillamine and trientine, respectively (Fig 3A). Finally, after excluding PT signals related to product quality, use issues, and drug indications, 69 and 34 PT signals were obtained for penicillamine and trientine, respectively. All AE signals are presented in S1 Table.Our analysis focused on the top 30 AE signals with the highest frequency and signal intensity (Table 3; Fig 3B). The three most common AE signals for penicillamine were drug hypersensitivity,condition aggravated, and dystonia, while elastosis perforans (PRR = 56046.64), pseudoxanthoma elasticum (PRR = 8622.56), and skin degenerative disorder (PRR = 2030.68) were the three most common AE signals with the highest PRR values. On the other hand, the most commonly reported AEs associated with trientine were tremors, abdominal pain, and condition aggravated, while starvation (PRR = 340.48), neurological symptom (PRR = 70.17), and oesophageal ulcer (PRR = 60.72) were the three AE signals with the highest PRR values.

**Table 3:** Comparison of the first 30 high signal intensity adverse events (AEs) between penicillamine and trientine.

NO	Penicillamine					Trientine				
	PT	n	ROR (95% CI)	PRR (X^2^)	IC (IC025)	PT	n	ROR (95% CI)	PRR (X^2^)	IC (IC025)
**1**	Drug hypersensitivity	37	7.89 (5.69 - 10.94)	7.71 (216.89)	2.95 (2.47)	Tremor	12	5.71 (3.23 - 10.1)	5.63 (45.86)	2.49 (1.69)
2	Hypersensitivity	22	4.98 (3.27 - 7.59)	4.92 (68.95)	2.3 (1.69)	Abdominal pain	10	3.45 (1.85 - 6.44)	3.42 (17.19)	1.77 (0.9)
3	Condition aggravated	19	2.74 (1.74 - 4.3)	2.71 (20.64)	1.44 (0.79)	Condition aggravated	10	2.75 (1.47 - 5.13)	2.73 (10.99)	1.45 (0.57)
4	Dystonia	13	26.96 (15.61 - 46.56)	26.73 (321.85)	4.74 (3.96)	Depression	8	2.72 (1.36 - 5.46)	2.7 (8.61)	1.43 (0.47)
5	Foetal exposure during pregnancy	13	6.72 (3.89 - 11.6)	6.67 (62.72)	2.74 (1.96)	Anxiety	8	2.2 (1.1 - 4.41)	2.19 (5.17)	1.13 (0.16)
6	Elastosis perforans	12	56513.69 (23066.46 - 138460.66)	56046.64 (269014.38)	14.45 (13.43)	Memory impairment	7	4.01 (1.91 - 8.44)	3.98 (15.68)	1.99 (0.97)
7	Cutis laxa	10	1504.93 (798.17 - 2837.53)	1494.58 (14351.78)	10.49 (9.6)	Dysphagia	7	5.95 (2.83 - 12.53)	5.91 (28.58)	2.56 (1.54)
8	Anti-neutrophil cytoplasmic antibody positive vasculitis	10	189.25 (101.45 - 353.03)	187.95 (1850.31)	7.55 (6.67)	Constipation	7	2.66 (1.27 - 5.61)	2.65 (7.2)	1.4 (0.38)
9	Exposure during pregnancy	10	6.96 (3.74 - 12.97)	6.92 (50.72)	2.79 (1.92)	Abdominal discomfort	6	2.89 (1.29 - 6.45)	2.87 (7.34)	1.52 (0.43)
10	Glomerulonephritis rapidly progressive	9	351.74 (182.09 - 679.46)	349.56 (3099.16)	8.44 (7.52)	Dyspepsia	6	4.95 (2.22 - 11.05)	4.92 (18.76)	2.3 (1.2)
11	Liver disorder	7	6.62 (3.15 - 13.91)	6.59 (33.21)	2.72 (1.7)	Abdominal pain upper	6	2.35 (1.05 - 5.25)	2.34 (4.62)	1.23 (0.13)
12	Hepatic cirrhosis	7	16.07 (7.65 - 33.78)	16 (98.43)	4 (2.98)	Acute kidney injury	5	2.67 (1.11 - 6.42)	2.66 (5.17)	1.41 (0.23)
13	Liver transplant	7	81.29 (38.65 - 170.95)	80.9 (551.23)	6.33 (5.31)	Neurological symptom	5	70.62 (29.3 - 170.24)	70.17 (340.6)	6.13 (4.95)
14	Pulmonary alveolar haemorrhage	7	53.41 (25.4 - 112.3)	53.16 (357.78)	5.73 (4.71)	Cognitive disorder	5	8.61 (3.57 - 20.74)	8.56 (33.4)	3.1 (1.91)
15	Nephrotic syndrome	7	42.89 (20.4 - 90.17)	42.69 (284.69)	5.41 (4.39)	Gastrooesophageal reflux disease	4	4.02 (1.51 - 10.75)	4.01 (9.04)	2 (0.71)
16	Pleural effusion	7	4.7 (2.24 - 9.88)	4.68 (20.29)	2.23 (1.2)	Seizure	4	2.88 (1.08 - 7.69)	2.87 (4.88)	1.52 (0.23)
17	Drug intolerance	6	2.64 (1.18 - 5.88)	2.63 (6.07)	1.39 (0.3)	Stress	4	4.49 (1.68 - 12)	4.47 (10.8)	2.16 (0.87)
18	Dysarthria	6	6.56 (2.94 - 14.62)	6.53 (28.14)	2.71 (1.61)	Hepatic cirrhosis	4	17.55 (6.57 - 46.9)	17.47 (62.1)	4.13 (2.83)
19	Dysphagia	6	2.66 (1.19 - 5.92)	2.65 (6.17)	1.41 (0.31)	Colitis ulcerative	4	7.73 (2.89 - 20.66)	7.7 (23.32)	2.94 (1.65)
20	Vasculitis	6	21.37 (9.58 - 47.66)	21.29 (115.97)	4.41 (3.32)	Dysarthria	4	8.36 (3.13 - 22.34)	8.32 (25.78)	3.06 (1.76)
21	Gastrointestinal disorder	6	3.03 (1.36 - 6.76)	3.02 (8.13)	1.6 (0.5)	Hepatic failure	4	10.42 (3.9 - 27.85)	10.37 (33.89)	3.37 (2.08)
22	Skin degenerative disorder	5	2037.69 (827.21 - 5019.51)	2030.68 (9620.54)	10.91 (9.7)	Aggression	4	6.18 (2.31 - 16.52)	6.16 (17.29)	2.62 (1.33)
23	Ageusia	5	8.26 (3.43 - 19.88)	8.24 (31.8)	3.04 (1.86)	Laboratory test abnormal	3	8.07 (2.6 - 25.07)	8.04 (18.49)	3.01 (1.56)
24	Renal impairment	5	2.51 (1.04 - 6.05)	2.51 (4.54)	1.33 (0.15)	Oesophageal ulcer	3	60.96 (19.61 - 189.52)	60.72 (176.07)	5.92 (4.48)
25	Nephrolithiasis	5	4.62 (1.92 - 11.13)	4.61 (14.15)	2.21 (1.02)	Dyskinesia	3	5.81 (1.87 - 18.05)	5.79 (11.89)	2.53 (1.09)
26	Pancytopenia	5	3.77 (1.57 - 9.08)	3.76 (10.15)	1.91 (0.73)	Gallbladder disorder	3	13.09 (4.21 - 40.67)	13.04 (33.35)	3.7 (2.26)
27	Anaphylactic reaction	5	3.94 (1.64 - 9.48)	3.93 (10.92)	1.97 (0.79)	Ill-defined disorder	3	3.9 (1.26 - 12.13)	3.89 (6.45)	1.96 (0.51)
28	Skin disorder	5	6.33 (2.63 - 15.23)	6.31 (22.35)	2.66 (1.48)	Liver disorder	3	5.41 (1.74 - 16.82)	5.4 (10.75)	2.43 (0.99)
29	Inguinal hernia	5	33.61 (13.96 - 80.91)	33.5 (157.52)	5.06 (3.88)	Burning sensation	3	3.37 (1.08 - 10.46)	3.36 (4.97)	1.75 (0.3)
30	Dementia	4	6.2 (2.32 - 16.54)	6.18 (17.39)	2.63 (1.34)	Gastric ulcer	3	12.31 (3.96 - 38.24)	12.26 (31.03)	3.62 (2.17)

The results showed that the top-ranked AEs for both drugs were generally consistent with those listed in the drug prescribing information. In addition, several AEs not documented in the penicillamine labeling were identified, including cirrhosis [n = 7, ROR (95% CI) 16.07 (7.65–33.78)], pleural effusion [n = 7, ROR (95% CI) 4.7 (2.24–9.88)], cerebral atrophy [n = 4, ROR (95% CI) 39.76 (14.89–106.14)], herpetic dermatitis [n = 4, ROR(95% CI) 23.27 (8.72–62.09)], pemphigus [n = 4, ROR(95% CI) 14.69 (5.5–39.2)], Stevens-Johnson Syndrome [n = 4, ROR(95% CI) 7.33 (2.75–19.57)], dementia [n = 4, ROR(95% CI) 6.2 (2.32–16.54)], torticollis [n = 3, ROR(95% CI) 59.52 (19.16–184.94)],positive antinuclear antibodies [n = 3, ROR(95% CI) 36.65 (11.8–113.82)], drooling [n = 3, ROR(95% CI) 18.66 (6.01–57.95)], atrial septal defect [n = 3, ROR(95% CI) 14.12 (4.55–43.85)], respiratory distress [n = 3, ROR (95% CI) 4.48 (1.44–13.92)], hepatic failure [n = 3, ROR (95% CI) 4.08 (1.31–12.66)], and decreased bone density [n = 3, ROR (95% CI) 3.74 (1.21–11.62)], and stronger-signal but lower-frequency AEs were also observed, including decreased blood copper [n = 3, ROR(95% CI) 1755.08 (550.76–5592.83)], copper deficiency [n = 3, ROR (95% CI) 1182.37 (374.2–3735.98)], SJS-TEN overlap [n = 3, ROR (95% CI) 247.96 (79.58–772.61)], micrognathia [n = 3, ROR(95% CI) 231.12 (74.19–719.96)], congenital central nervous system anomaly [n = 3, ROR(95% CI) 231.12 (74.19–719.96)]. In contrast, adverse events not documented in the labeling of trientine, including tremor [n = 12, ROR (95% CI) 5.71 (3.23–10.1)], memory impairment [n = 7, ROR (95% CI) 4.01 (1.91–8.44)], and dysphagia [n = 7, ROR(95% CI) 5.95 (2.83–12.53)], acute kidney injury [n = 5, ROR(95% CI) 2.67 (1.11–6.42)], cognitive impairment [n = 5, ROR(95% CI) 8.61 (3.57–20.74)], epileptic seizures [n = 4, ROR(95% CI) 2.88 (1.08–7.69)], stress [n = 4, ROR(95% CI) 4.49 (1.68–12)], dysarthria [n = 4, ROR (95% CI) 8.36 (3.13–22.34)], esophageal ulceration [n = 3, ROR (95% CI) 60.96 (19.61–189.52)], gallbladder lesions [n = 3, ROR (95% CI) 13.09 (4.21–40.67)], burning sensation [n = 3, ROR (95% CI) 3.37 (1.08–10.46)], gastric ulcers [n = 3, ROR (95% CI) 12.31 (3.96–38.24)], and starvation [n = 3, ROR (95% CI) 341.82 (109.7–1065.1)]. These findings suggest potential new safety signals that need to be further investigated, whereas hepatic system-related AEs (cirrhosis, liver failure) may reflect indirect consequences of copper metabolism disorders and need to be combined with clinical monitoring. Although the signals of penicillamine-related cerebral atrophy (n = 4) and trientine-related starvation (n = 3) are of high intensity, the number of cases is small, and validation through prospective studies combined with blood copper and imaging data is needed.

### Correlations of major AE signal tests with SOCs

**Fig 4:**
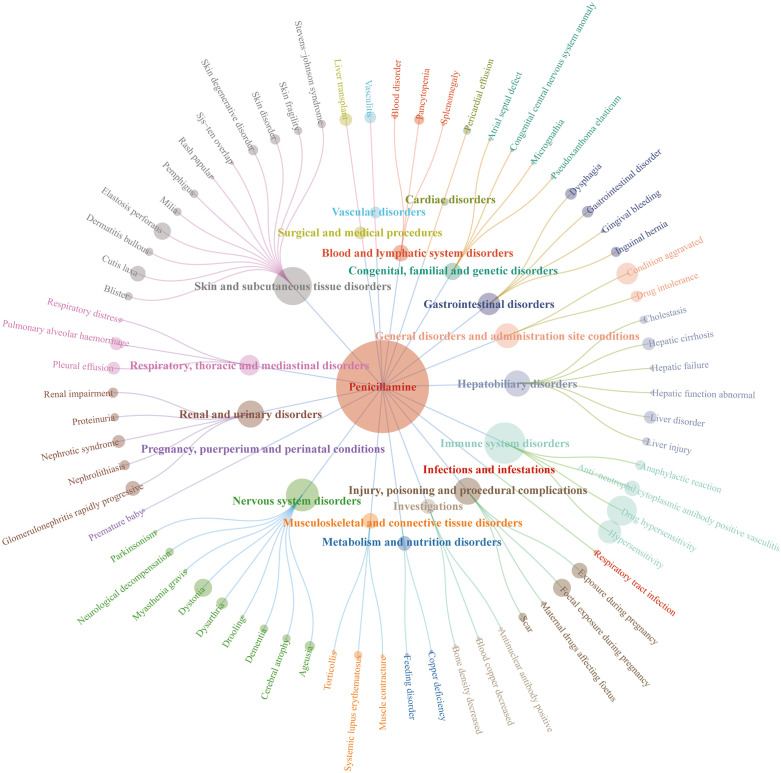
Network plot of the distribution of AE signals in each SOC of penicillamine. The root node represents the drug name and the number of AE signals, the inner circle is the SOC, and the outer circle is the AE signal name.See S2 Fig for Figure trientine.

**Fig 5:**
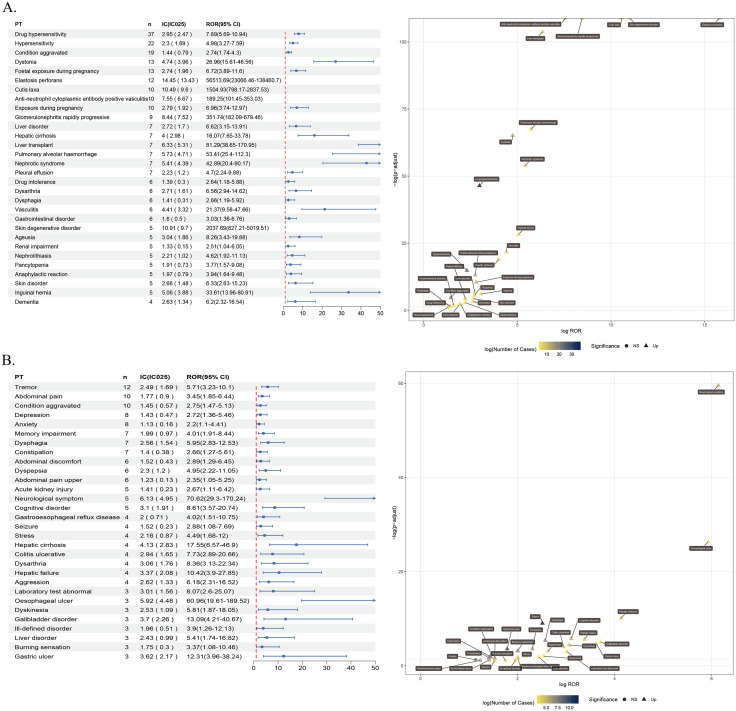
Forest plot and volcano plot of ROR values (95% CI) for the first 30 high signal intensity AEs of penicillamine versus trientine. A,penicillamine;B,trientine.Two drugs-related AE volcano plots;ROR,reporting odds ratio; P-adjust, p-value after Bonferroni correction.

The two drugs’ AE signals were classified according to SOCs using MedDRA 27.0. Significant differences were observed in AE signals related to penicillamine and trientine in organ system distribution (Fig 4, Table 4). Penicillamine-related AE signals mainly revolved around immune system (18.41%), skin and subcutaneous tissue (15.42%), and neurological (11.19%) disorders. Conversely, those of trientine predominantly featured GI (32.94%), neurological (27.06%), and psychiatric (14.12%) disorders. Notably, the distribution characteristics aligned with the mechanisms of action of both drugs and previous clinical observations, further confirming the heterogeneity of their safety profiles. We further determined the ROR 95% Confidence Intervals (CIs) of signal intensities for the top 30 AEs (Table 3) with the highest frequency of occurrence for both drugs. The results were visualized using forest plot and volcano plots(Fig 5).In each volcano plot, the x-axis indicates the logarithm of the ROR. The y-axis represents the negative logarithm of the p-adjust from the p-value after the Fisher’s exact test and Bonferroni correction. A positive y-axis represents a strongly significant difference. The color of the dot indicates the logarithm of the number of case reports. The darker the color, the higher the number of reports. Thus, drugs in the upper right of the graph had both significant signal strength and differences.

**Table 4:** Comparison of system organ classification (SOC) Distribution of adverse events between penicillamine and trientine.

SOC	Penicillamine	Trientine
	n(%)	n(%)
Immune system disorders	74(18.41)	0(0)
Skin and subcutaneous tissue disorders	62(15.42)	0(0)
Nervous system disorders	45(11.19)	46(27.06)
Renal and urinary disorders	30(7.46)	5(2.94)
Injury, poisoning and procedural complications	30(7.46)	0(0)
Hepatobiliary disorders	28(6.97)	17(10.00)
General disorders and administration site conditions	25(6.22)	13(7.65)
Gastrointestinal disorders	20(4.98)	56(32.94)
Respiratory, thoracic and mediastinal disorders	17(4.23)	0(0)
Congenital, familial and genetic disorders	12(2.99)	0(0)
Blood and lymphatic system disorders	11(2.74)	0(0)
Musculoskeletal and connective tissue disorders	10(2.49)	3(1.76)
Investigations	9(2.24)	3(1.76)
Surgical and medical procedures	7(1.74)	0(0)
Vascular disorders	6(1.49)	0(0)
Metabolism and nutrition disorders	6(1.49)	3(1.76)
Cardiac disorders	4(1.00)	0(0)
Pregnancy, puerperium and perinatal conditions	3(0.75)	0(0)
Infections and infestations	3(0.75)	0(0)
Psychiatric disorders	0(0)	24(14.12)

For penicillamine-related immune system disorders, drug hypersensitivity reactions (n = 37, ROR = 7.89 [5.69–10.94]) and Anti-Neutrophil Cytoplasmic Antibody (ANCA)-positive vasculitis (n = 10, ROR = 189.25 [101.45–353.03]) were the core risk events (Table 3). Such high signal intensity events could be attributed sulfhydryl structure-induced immunogenic responses. Among penicillamine-related skin and subcutaneous tissue disorders, perforating lesions of elastic tissue (n = 12, PRR = 56513.69 [23066.46–138460.66]) and skin laxity (n = 10, PRR = 1504.93 ([798.17–2837.53]) were particularly prominent in terms of signal intensity. Based on these findings, we hypothesized that penicillamine may either interfere with collagen metabolism or directly damage skin elastic fibers, leading to irreversible structural damage. For neurological disorders, we found a high frequency of dystonia (n = 13, ROR = 26.96 ([15.61–46.56]) and dysarthria (n = 6, ROR = 6.56 [2.94–14.62]), attributable to a copper depletion-induced neurotransmitter imbalance or neurotoxicity resulting from a direct drug penetration of the Blood-Brain Barrier (BBB). Although penicillamine’s congenital anomalies (2.99%) accounted for a relatively small percentage of cases, their signal strength was extremely high. For instance, both elastic pseudoxanthoma (ROR = 8640.41 [2459.58–30353.45]) and micrognathia (ROR = 231.12 ([74.19–719.96]) had RORs > 200, and their potential teratogenic risks were to be rigorously evaluated in females of childbearing age. On the other hand, GI disorders (32.94%) dominated the AE signals for trientine, with abdominal pain (n = 10, ROR = 3.45 ([1.85–6.44]), dysphagia (n = 7, ROR = 5.95 [2.83–12.53]), and constipation (n = 7, ROR = 2.66 ([1.27–5.61]) as the main events. These AEs could be attributed to direct irritation of the GI mucosa by the drug or disturbed GI motility following copper chelation. For neurological disorders, tremors (27.06%) had the highest frequency (n = 12, ROR = 5.71 [3.23–10.1]). Notably, this signal has been associated with a copper homeostasis imbalance in the brain following trientine-promoted copper excretion via feces. Consequently, it is predisposed to inducing symptomatic fluctuations in patients with severe baseline neurological copper accumulation. Although the signal intensities associated with the high percentage of psychiatric events (14.12%) in depression (n = 8, ROR = 2.72 [1.36–5.46]) and anxiety (n = 8, ROR = 2.2 [1.1–4.41]) were low, its suggests that patients with a history of comorbid psychiatric disorders may experience significant challenges regarding treatment adherence. Among hepatobiliary system disorders (10.00%), cirrhosis showed a significant signal intensity (n = 4, ROR = 17.55 [6.57–46.9]), implying that long-term medication may exacerbate the risk of hepatic injury via copper redistribution or drug accumulation. Notably, the AE with the highest signal intensity for trientine was starvation (n = 3, ROR = 341.82 [109.7–1065.1]), and although the possible mechanism has been associated with metabolic disturbances, it remains unclear.

### SOC comparisons

**Fig 6:**
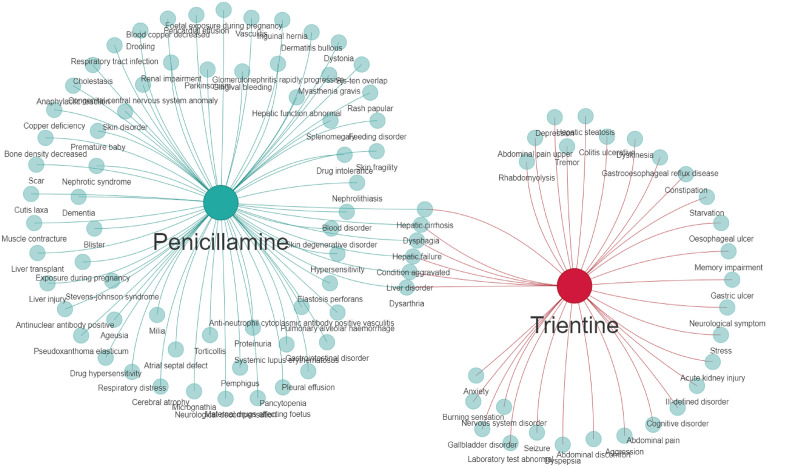
Wayne diagram showing the PT positive signaling network of two copper chelators in the FAERS database.

We also observed differences in SOC distribution between the two drugs. Specifically, penicillamine’s AE profile was more skewed towards immune-mediated and dermal toxicity, whereas that of trientine was inclined towards GI disorders and neurotoxicity. Compared to trientine, penicillamine accounted for a significantly higher proportion of immune system diseases (0% vs. 18.41%). Furthermore, the intensities of its signals such as degenerative skin diseases were much higher than those of trientine. Conversely, the proportion of GI diseases related to trientine was 6.6-fold higher than penicillamine’s (32.94% vs. 4.98%), with signals such as esophageal ulcers (ROR = 60.96 [19.61–189.52]) suggesting a more serious risk of GI injury. Six AEs were common between the two drugs, including dysphagia, hepatic failure, aggravated condition, liver disorder, hepatic cirrhosis, and dysarthria (Fig 6). Most of these AEs involved the hepatobiliary system and were reported in 6.97% and 10.0% of penicillamine and trientine cases, respectively. Besides the events related to the hepatobiliary system, penicillamine’s nephrotoxicity of (7.46%) and trientine’s Acute Kidney Injury (n = 5, ROR = 2.67 [1.11–6.42}) further suggested that both classes of drugs could affect renal function, highlighting the need to closely monitor patients’ renal function during clinical application for individualized dosing.

### Time to onset and Weibull distribution analysis of AEs

**Fig 7:**
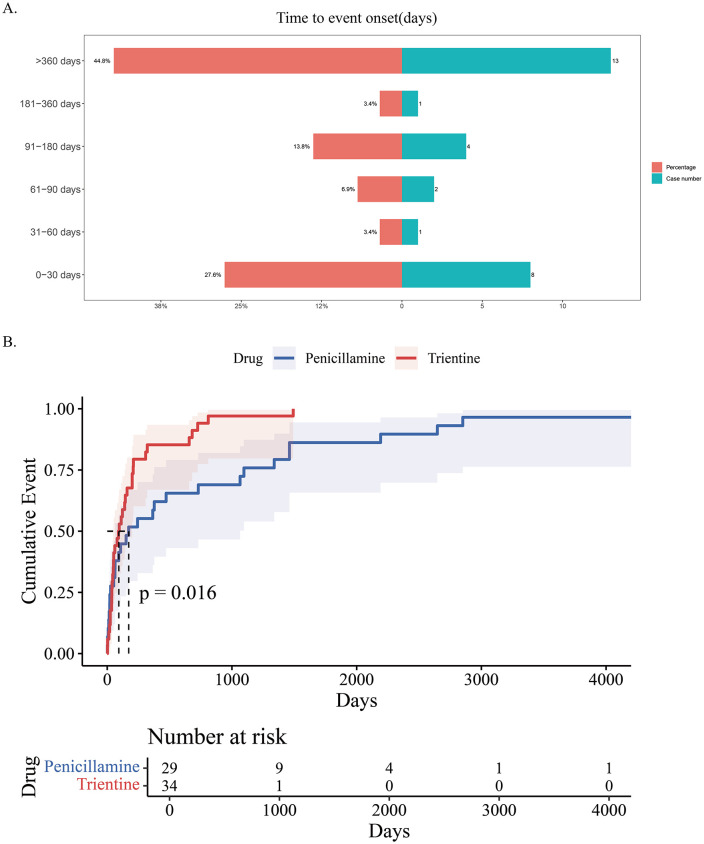
A Time to onset of adverse events induced by penicillamine. B Cumulative incidence of adverse events related to two drugs over time. See S3 Fig for Figure **C.**

We used the interquartile range (IQR) method to detect and exclude outliers in the time-to-onset of adverse drug events in the two groups. TTO analysis based on the Weibull distribution model indicated that both penicillamine and trientine exhibited an early failure pattern (shape parameter β < 1) in inducing AEs, but the two drugs showed significant differences in their temporal characteristics. Trientine had a significantly earlier median time to onset (TTO) than penicillamine, with a more concentrated temporal distribution, suggesting that its AE may manifest more rapidly in the early stages of treatment. Penicillamine not only had a higher early risk (β = 0.55 vs 0.76) but also exhibited a gradual increase in risk over time and a broader time span for onset, indicating that long-term use requires continued vigilance. Fig 7 clearly illustrates the temporal distribution of these events. Cumulative incidence curves and temporal distributions are shown in Fig 7B and Table 5. However, the conclusions of this study are limited by the small sample size (n = 29 in the penicillamine group and n = 34 in the trientine group), which may result in wider confidence intervals for parameter estimates. Additionally, the study did not account for confounding factors such as the severity of underlying diseases or concomitant medications, which may affect the generalizability of the findings. Further studies with larger sample sizes are needed to validate the temporal distribution characteristics.

**Table 5:** Time to onset of -associated adverse events and Weibull distribution analysis.

Drug	Case number	TTO(days)	Weibull distribution		
		Median (IQR)	Scale parameter:α(95%CI)	Shape parameter:β(95%CI)	Type
Penicillamine	29	172(24-1218)	453.17(137.17-769.16)	0.55(0.39-0.71)	Early failure
Trientine	34	93(37-208)	177.20(94.14-260.25)	0.76(0.57-0.95)	Early failure

Abbreviation: TTO,time to onset; CI, confidence interval; IQR, interquartile range.

### Gender differences in risk signals for penicillamine

**Fig 8:**
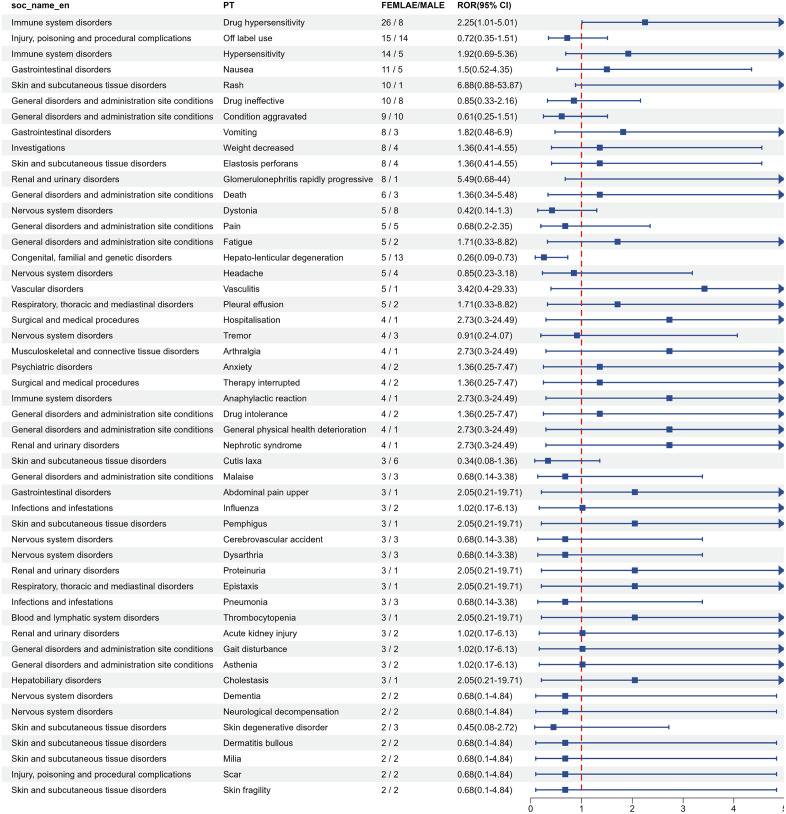
Forest plot of ROR values used to assess gender differences in penicillamine-related AEs.

Based on the demographics of the two groups of drug-related AEs from the FAERS database (Table 2), penicillamine’s gender distribution showed a significantly higher proportion of female reports than male reports. Meanwhile, trientine showed a non-significant difference, with a male-to-female ratio of about 1:1.1. In addition, reports related to trientine had a high proportion of unknown gender (19.5%). Therefore, we performed a gender-based subgroup analysis only for penicillamine to explore the effect of gender on the risk of AEs.Only the top 50 AEs common to both males and females were identified using the ROR method and categorized by SOC (Fig 8). S2 Table shows the results for all data. Due to gender differences in determining all PT signals for penicillamine, we could not screen for other relevant PTs, such as product use problems and over-the-counter use, among others. Nonetheless, there were significant gender differences in drug hypersensitivity, hypersensitivity, nausea, rash, vomiting, weight decreased, elastosis perforans, glomerulonephritis rapidly progressive, fatigue, vasculitis,which were more common in women.Conversely, dystonia (n = 8, ROR = 0.42 [0.14–1.3]) and cutis laxa (n = 6, ROR = 0.34 [0.08–1.36]) were the high-risk AEs in males. Notably, The results of the study based on the analysis of the gender subgroups of penicillamine should be interpreted with caution, as the sample size was small and gender data were missing (8.8% for penicillamine and 19.5% for trientine), which may limit the generalizability of the results.

## Discussion

Penicillamine and trientine, copper chelators used in WD treatment, have been established to promote copper excretion, significantly improving clinical outcomes. Unlike the FAERS analysis by Kumar et al [14]. (1970–2020), which focused on a single drug, this study represents the first 20-year systematic comparison of penicillamine and trientine using three signal detection methods. We further identified potential signals not previously reported in cohort studies, more clearly delineating the adverse event profiles of both drugs.Specifically, we explored the risk profiles of their AEs using different analytical methods and systematically compared their safety profiles in relation to WD treatment. The number of penicillamine-related AEs (n = 1452) was ⁓1.9 times higher than that of trientine (n = 760). This phenomenon could be attributed to penicillamine’s longer history of clinical use and wider prescription volume. Notably, trientine, as a second-line drug, exhibited a significant increase in clinical use in recent years (44.8% share in 2022–2024). Despite the gradual increase in its use, it has not yet completely replaced penicillamine. Regarding gender distribution, penicillamine was significantly more commonly reported in females (54.5%) than in males (36.7%)—a phenomenon that aligns with the literature that reported females being more likely to experience AEs [26]. Conversely, trientine showed a smaller gender difference (42.4% in females; 38.0% in males), implying its lower gender sensitivity. In clinical applications, the AEs of both drugs involved multiple SOCs, including the immune system, nervous system, gastrointestinal system, and hepatobiliary system. Penicillamine was dominated by Immune system disorders (18.41%), while trientine focused on Gastrointestinal disorders (32.94%). Particularly, some of the AEs of trientine involved Psychiatric disorders (14.12%), which was not mentioned in the drug labeling instructions of trientine. This study identified all the AEs listed in the drug labeling instructions and found six common AEs for both drugs, including condition aggravated.The hepatobiliary system category accounted for a higher proportion of these AEs, with hepatic cirrhosis, hepatic failure, and liver disorder as the key AE.

Penicillamine, a pioneer copper chelator, could be traced back to the 1950s, when Walshe first used it for WD treatment based on his casual observations of copper excretion in cystinuria patients [5]. Despite its good efficacy in WD treatment, especially in acute copper exclusion, penicillamine has demonstrated a high incidence of drug sensitization reactions and multisystemic AEs, prompting the development of new-generation copper chelators. Consequently, trientine was developed and approved in 1969 as a polyamine derivative. Its molecular structure includes an ethylenediamine moiety linked to a tetramine chelation site, enhancing its selectivity for divalent copperions [8].

In this study, penicillamine (18.41%) exhibited a significantly higher incidence of immune-related AEs than trientine. According to research, penicillamine may irreversibly bind to aldehyde groups on the surface of macrophages, inducing autoimmune reactions, which in turn, activate macrophages and prompt them to release pro-inflammatory factors such as Tumor Necrosis Factor-alpha (TNF-α), IL-6, and IL-23. These factors would then trigger a positive feedback activation of Natural killer (NK) cells and T cells, as well as the exposure of self-antigens [27–29]. This dual-action mechanism could theoretically explain the observed autoimmune events such as drug hypersensitivity (ROR = 7.89) and ANCA-positive vasculitis (ROR = 189.25).There have also been several case reports of penicillamine-induced autoimmune events during clinical use. For instance, Rozina et al [30] reported four WD patients (three females and one male) who developed significant immune system disorders, including Interstitial Lung Disease (ILD), drug-induced lupus, or ANCA-associated vasculitis, while under penicillamine medication. Besides autoimmune AEs, we also explored the penicillamine-induced skin and subcutaneous tissue disorders. Elastosis perforans (ROR = 56,513.69) and cutis laxa (ROR = 1,504.93) both exhibited extremely high signal intensities. The mechanism may disrupt extracellular matrix homeostasis through multiple pathways, including indirect inhibition of lysyl oxidase (LO) activity via copper ion binding, disturbance of elastic fiber metabolism, and disorders of collagen metabolism.ultimately causing skin elastic tissue lesions [31–33], clinical manifestations of Elastosis Perforans Serpiginosa(EPS), and skin laxity, among other effects. Despite a low incidence, we also found that the ROR value of penicillamine’s congenital anomaly signal was high (e.g., micrognathia ROR = 231.12), implying that women of childbearing age may have to be evaluated for potential teratogenic risk. Nonetheless, the administration of copper chelation therapy during pregnancy in WD patients remains controversial. While earlier studies recommended that penicillamine should not be administered to WD patients during pregnancy [34], more recent relevant studies have disproved this view [35,36]. According to the 2025 EASL guidelines, the benefits of anti-copper therapy in pregnant WD patients are significantly greater than the teratogenicity risk the drug poses to the fetus. Furthermore, female WD patients who wish to become pregnant or who are already pregnant should be monitored closely to support anti-copper drug therapy [6].

Although trientine avoids the associated toxicity, its potent copper chelating effect may cause a sudden drop in copper ions within the intestinal mucosa. This reduces the antioxidant capacity of the intestinal mucosa (e.g., decreased SOD1 activity), increases the risk of oxidative damage, impairs local redox balance, and thereby induces inflammation and ulceration [37]. This phenomenon could explain the GI system AEs (e.g., oesophageal ulcer ROR = 60.96, abdominal pain ROR = 3.45) associated with trientine in this study. Besides neurologic AEs such as tremors (ROR = 5.71) and cognitive disorder (ROR = 8.61), trientine could also cause drug-induced neurologic deterioration. Generally, copper chelation therapy (e.g., penicillamine, trientine) could induce neurologic deterioration in WD patients who have a high baseline brain copper load. Członkowska et al [38] reported early deterioration in 35% of neurotypical patients in the D-penicillamine-treated group, possibly due to rapid copper redistribution. Neurologic deterioration can either be “primary” or “secondary” to drug-induced deterioration in WD patients [39] and should be differentiated based on the clinical situation. Notably, we found that the “starvation” signal (ROR = 341.82) of trientine was extremely strong. Although the underlying mechanism remains unclear, we speculated that copper chelators may interfere with metal ion homeostasis in the hypothalamic feeding center and affect leptin and neuropeptide Y secretion, thus inducing starvation. Psychiatric events associated with trientine (such as depression and anxiety) accounted for 14.12% of reported cases. As copper serves as a cofactor for tyrosine hydroxylase (TH), its depletion may reduce dopamine synthesis, leading to mood abnormalities [40]. However, whether psychiatric disorders occurring in WD patients constitute drug-induced adverse events requires differentiation based on actual clinical circumstances.

Penicillamine and trientine also exhibited common hepatobiliary AEs, with cirrhosis (ROR = 16.07 and 17.55, respectively) and liver failure (ROR = 4.08 and 5.41, respectively) as the most common events. Long-term copper chelation therapy may lead to copper redistribution or drug accumulation, thus aggravating liver injury. Consequently, liver function monitoring is key to long-term management regardless of the chelating agent selected. Moreover, the prevalence of penicillamine-related nephrotoxicity (7.46%) and the elevated risk of trientine-associated AKI (ROR = 2.67) suggest that both drugs may affect renal function. Through the immune complex deposition mechanism, penicillamine could induce membranous nephropathy or even nephrotic syndrome. Furthermore, although the nephrotoxicity associated with trientine is relatively low, clinicians should still watch out for the risk of tubulointerstitial injury. In this regard, treatment regimens should be clinically adjusted according to renal function indexes, with preference given to zinc agents when necessary [41]. We also explored gender differences in penicillamine-related AEs based on real-world data and found that female patients were more prone to immune system disorders, such as drug hypersensitivity, ANCA-positive vasculitis, and skin toxicities (such as rash and elastosis perforans). On the other hand, male patients were more prone to neurologic events (e.g., dystonia) and degenerative skin disorders (e.g., cutis laxa). This difference could be attributed to the hyperresponsiveness of the female immune system. According to research, estrogen enhances B-cell activity and promotes antibody production, thus increasing the risk of immune-related AEs [42]. Moreover, the nervous system disorders observed in male patients may be related to penicillamine-induced elevation of free copper in the brain and enhanced oxidative stress. Conversely, the gender difference in AEs for trientine was not significant, possibly due to lower immunogenicity and a more homogeneous tissue distribution.While penicillamine showed higher hypersensitivity in females, this observation requires validation in prospective cohorts due to potential reporting bias.

### Limitations

Despite its valuable insights, this study had several notable limitations. First, the inherent limitations of FAERS data—the rate of false reporting was unknown, especially mild AEs; Unable to establish a causal relationship, confounding factors (such as concomitant medications) may affect the signal; There is a lack of data on dose, course of treatment and underlying diseases, making it difficult to analyze the dose-effect relationship. For example, high doses of penicillamine may increase the risk of allergy, but this relationship cannot be quantified in this analysis; Furthermore, data duplication is processed by CASEID/FDA_DT screening, but residual duplicate records may still be present. Second, there was a significant percentage of unknown gender cases (8.8% for penicillamine and 19.5% for trientine), potentially affecting the accuracy of the gender difference analysis.Third,the relatively small sample size for trientine use could have led to the underestimation of the incidence of rare AEs. Fourth,“notoriety bias” caused by media coverage.Notably, future prospective cohort studies incorporating blood copper levels, genotyping, and imaging data will be required to further validate the biological mechanisms and risk factors for AEs associated with penicillamine and trientine.

## Conclusion

Penicillamine and trientine differ significantly in safety profiles, highlighting the need to weigh clinical drug selection for WD treatment against patients’ organ-specific risk. Trientine may be more suitable for women of childbearing age due to fewer immune-related AEs, but this hypothesis requires validation, while patients with poor gastrointestinal tolerance may need to prioritize penicillamine, though specific medication decisions should be based on actual clinical conditions.Nonetheless, more targeted copper chelators should be developed to better maintain the pharmacotherapeutic efficacy while circumventing the toxicity risks of existing drugs.

## Supporting information

S1 Fig Top 30 AE names and corresponding SOCs with the highest percentage of signals for trientine. (TIF)

S2 Fig Network plot of the distribution of AE signals in each SOC of trientine. (TIF)

S3 Fig Time to onset of adverse events induced by trientine. (TIF)

S1 Table PT and SOC for the positive signal detection of two drugs in the FAERS database. (XLSX)

S2 Table All Adverse Event Signals Screened by Penicillamine Gender Subgroup Analysis (FAERS). (XLSX)
