# Supplementary figures and images for: Safety of penicillamine and trientine in the treatment of Wilson’s disease: An analysis of the FDA Adverse Event Reporting System (FAERS) database
Source: PLoS One. 2025 Nov 12;20(11):e0336721. doi: 10.1371/journal.pone.0336721 (PMC12611112; doi:10.1371/journal.pone.0336721)

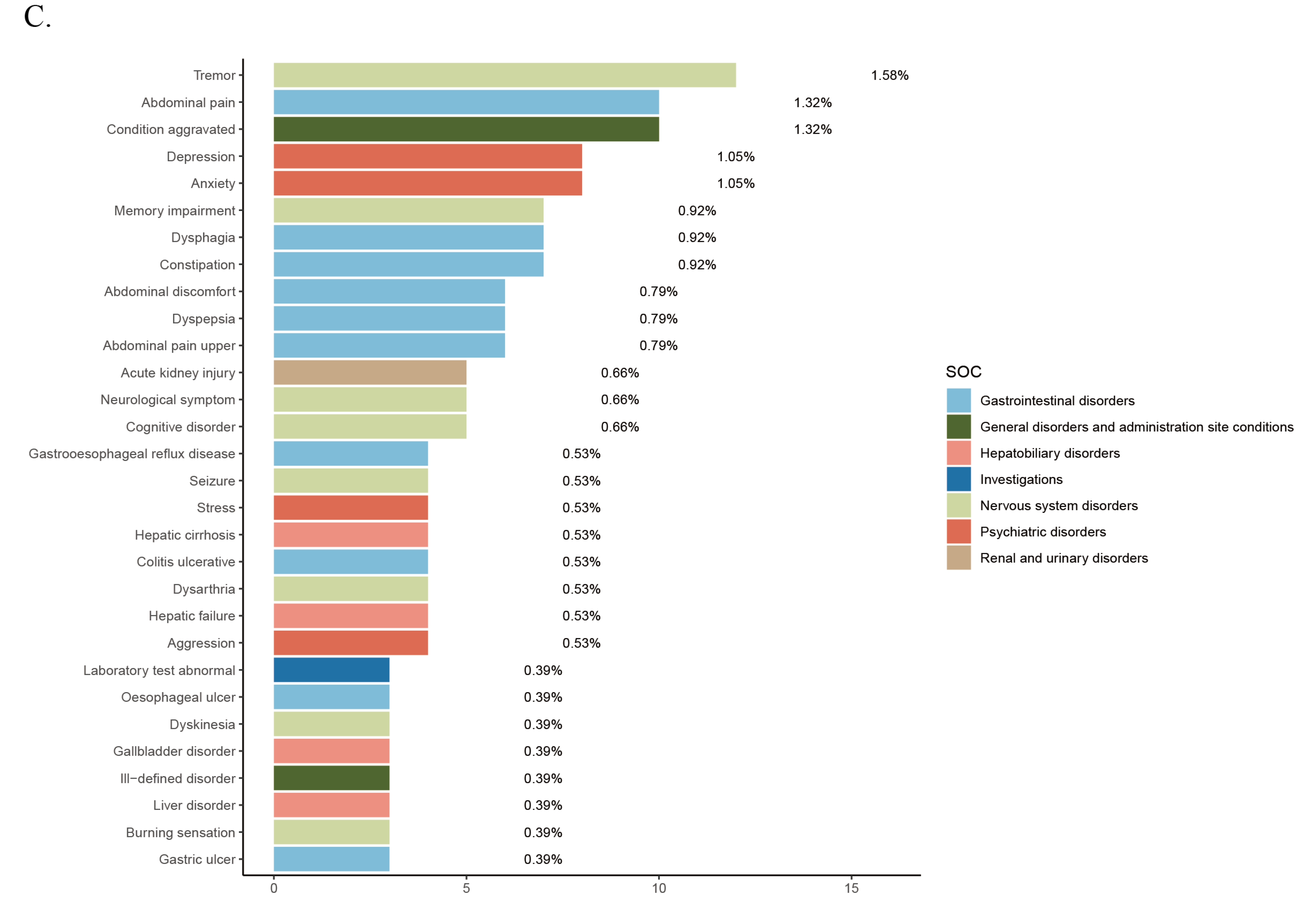

Supplement: S1 Fig — (TIF) [file pone.0336721.s001.tif]

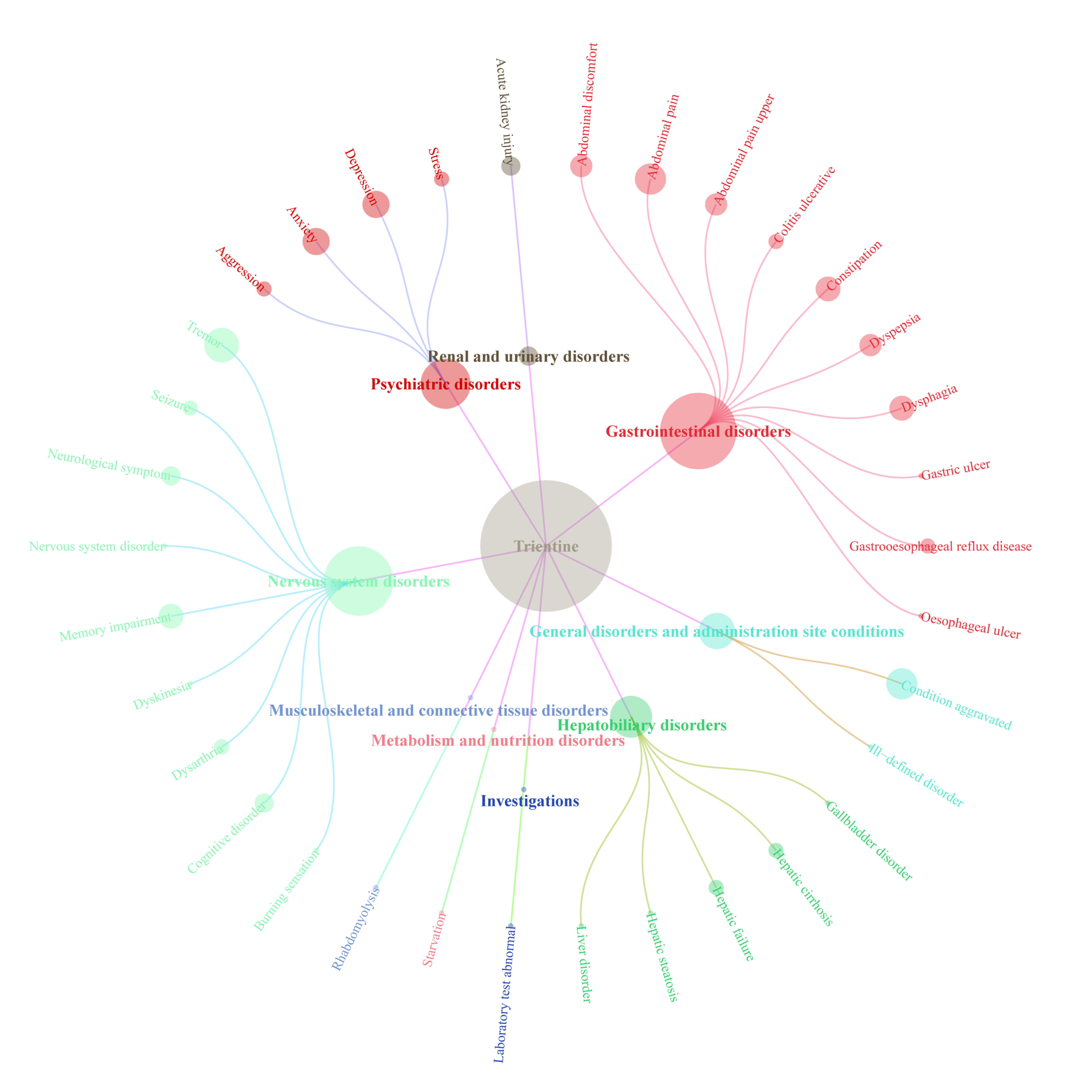

Supplement: S2 Fig — (TIF) [file pone.0336721.s002.tif]

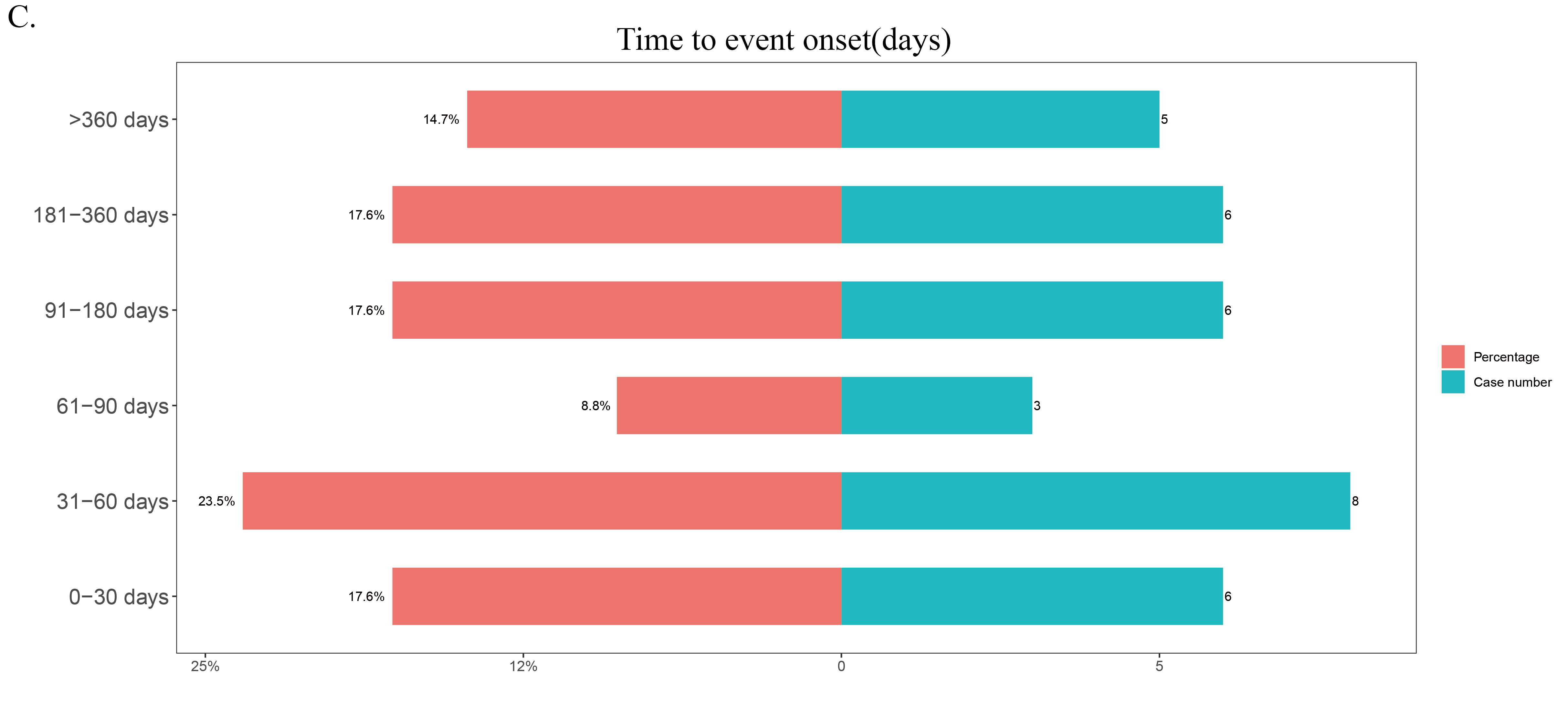

Supplement: S3 Fig — (TIF) [file pone.0336721.s003.tif]
